# Adolescent Mental Health: Impact of Introducing Earlier Compulsory School Grades

**DOI:** 10.1002/hec.4982

**Published:** 2025-07-09

**Authors:** Anna Linder, Ulf‐G. Gerdtham, Gawain Heckley

**Affiliations:** ^1^ Department of Clinical Sciences Malmö Health Economics Unit Lund University Malmö Sweden; ^2^ Centre for Economic Demography Lund University Lund Sweden; ^3^ Department of Economics Lund University Lund Sweden

**Keywords:** education policy, human capital development, mental health, school grades

## Abstract

We examine how the earlier introduction of compulsory school grades affects the likelihood of receiving a mental disorder diagnosis among Swedish adolescents. We exploit a school reform that shifted the introduction of grades from grade 8 to grade 6, resulting in first exposure to grading at different ages between cohorts. Our results show that girls exposed to earlier grading are more likely to be diagnosed with internalizing disorders, such as depression and anxiety, by the end of compulsory school. This effect is particularly pronounced among students with low to moderate academic achievement. We also find suggestive evidence that both girls and boys exposed to earlier grading face an increased risk of being diagnosed with alcohol‐related disorders. These findings highlight that early exposure to grading may have unintended adverse effects on adolescent mental health. Education systems should acknowledge these potential risks and consider implementing complementary mental health support when revising grading policies.

## 1 Introduction

A general shift toward increased school accountability, characterized by the ranking of schools and students based on performance measures, has led to a growing use of high‐stakes testing, assessment, and grading in schools in Sweden and other high‐income countries (Figlio and Loeb 2011; Lingard et al. 2013; Lundahl et al. 2017). At the same time, labor markets have become more skill‐intensive, increasing the importance of academic performance for future opportunities. These shifts may have contributed to rising school‐related pressure and, in turn, worsening mental health among adolescents (Högberg 2021). However, while the increase in adolescent mental health problems is well documented in many high‐income countries, including Sweden (Potrebny et al. 2017; Bremberg and Dalman 2015), the effects of school grading systems, and the potential role of intensified assessment practices, have received limited empirical attention.

In this paper, we study the effect of earlier introduction of grades in Swedish schools on mental disorder diagnoses during adolescence. Before the reform, students received individual grades beginning in eighth grade (around 14 years of age). However, in the fall of 2012, this changed so that grades were introduced starting in sixth grade (around age 12). The children who entered seventh grade during the reform year received grades for the first time in that grade (around age 13). This creates a natural variation in the timing of first exposure to grading, which is effectively arbitrary, depending on whether a child was born before or after the calendar year‐end school entry cutoff.

To isolate the effect of the reform, we control for age effects across the cutoff, as well as school‐starting‐age effects identified in previous research (Fredriksson and Öckert 2014), using a Difference‐in‐Discontinuities setup (Grembi et al. 2016). We combine this identification strategy with detailed administrative data on inpatient and outpatient diagnoses for several mental disorders. By this approach, we differentiate the discontinuity observed in cohorts exposed to grades at a younger age from the discontinuity related only to school‐starting‐age effects. What remains is the causal effect of earlier grading on mental disorders.

Theoretical perspectives suggest that performance feedback and ability ranking can have age‐sensitive effects on mental health, particularly for lower‐achieving children who hold fixed beliefs about their abilities (Dweck 2006). For these students, early exposure to evaluative feedback may undermine self‐efficacy and increase vulnerability to stress, anxiety, and depression (Fuhrmann et al. 2015; Kendall and Hollon 2013). However, the literature on how the timing of grading affects health outcomes remains limited (Lundahl et al. 2016). The research most closely related to our study is Högberg et al. (2021) who found that several concurrent changes within the Swedish school system, including earlier grading, negatively impacted self‐reported health among adolescents. Yet, their analysis bundled multiple reforms, including new curricula, revised learning objectives, and introducing a new grading scale with a failing grade, making it difficult to isolate the effect of earlier grading and draw clear policy conclusions. A few studies have explored the effects of grading age on non‐health outcomes. Sjögren (2010) analyzed a 1980s Swedish reform that removed written grade reports in lower and middle school and found it reduced educational attainment among girls. Similarly, Klapp (2015) showed that the same reform led to lower final grades in compulsory school and a decreased likelihood of completing upper secondary education.

Several previous studies suggest that the effects of grading are not uniform but vary by gender, academic ability, and parental background (Sjögren 2010; Klapp 2015). This is consistent with broader research on test‐based assessments, which finds heterogeneous impacts across different groups (Bandiera et al. 2015; Dee and Jacob 2011; Whitney and Candelaria 2017). A recent Swedish study further supports this view, showing that students—particularly young women—who received arbitrarily inflated grades, that is, grades exceeding their actual skill level, were less likely to be diagnosed with depression or anxiety and less likely to be prescribed psychotropic medication (Linder et al. 2023). These findings suggest that girls and young women may be especially sensitive to performance evaluation and grading practices, with implications for both mental health and human capital accumulation.

Our results show that exposure to earlier grading is associated with an increased likelihood of being diagnosed with mental disorders by the end of compulsory school. Girls exposed to grading 1 year earlier are significantly more likely to be diagnosed with internalizing disorders, such as depression and anxiety. These effects are particularly pronounced among girls with low to moderate academic achievement, while no increase is observed among the lowest‐ and highest‐achieving groups. We find no comparable effects for boys in this category. However, both girls and boys exposed to earlier grading are more frequently diagnosed with alcohol‐related disorders, though these associations are less robust under alternative model specifications, especially for boys.

Our paper contributes to the growing literature on the consequences of performance feedback through grade‐based assessments. Using a credible identification strategy, we isolate the causal impact of a grading reform to examine how earlier exposure to compulsory school grades affects adolescent mental health. We provide new evidence that this policy increased the likelihood of clinically diagnosed mental disorders among adolescents. Beyond evaluating the effects of this specific reform, our findings also offer broader insights into whether the global trend toward increased performance monitoring in schools may be contributing to rising mental health challenges among young people. Additionally, our results may shed light on potential mechanisms behind the mental health gap that emerges between boys and girls during school age and often persists into adulthood, particularly in relation to internalizing disorders.

The remainder of the paper is structured as follows. In the next section, we outline the institutional and theoretical context. Sections three and four describe the data and empirical strategy. Section five presents the main results, followed by section six, where we explore heterogeneity in effects by student performance, migration background, and socioeconomic status. In section seven, we conduct robustness checks, including tests for manipulation, covariate balance, baseline model assumptions, and placebo tests. Finally, sections eight and nine offer a discussion and conclude the paper.

## 2 Institutional and Theoretical Context

### 2.1 The Swedish School System

In Sweden education is compulsory for 10 years. School entry is determined by the year in which a child is born, with most children beginning preschool class (year 0) in the calendar year they turn six.^1^ In any given school year group, the oldest will be those born first of January and the youngest will be born 31^st^ of December. The academic year runs from mid‐August until mid‐June. Compulsory school is divided into three stages: years 0–3, years 4–6, and years 7–9. While some schools offer continuity throughout all grades, it is common for students to change schools between stages, particularly when entering year 7. School choice is allowed, but admission is generally determined by proximity to the school and relative distance compared to other applicants.

Upon completion of compulsory schooling, approximately 98% of students continue to upper secondary school, which lasts three years (The Swedish National Agency for Education 2024). In upper‐secondary school, students can choose between academic or vocational programs, both of which are designed to provide eligibility for higher education. Admission to these programs is based on students' final grades from compulsory school. For students who do not meet the requirements for direct entry, there are multiple introductory programs available, including preparatory tracks for core subjects or Swedish language acquisition. Despite high enrollment, around one in four students do not complete upper secondary education (The Swedish National Agency for Education 2022).

Education in Sweden is publicly funded through a school voucher system, allowing both public and privately operated schools to receive government funding. The national government sets educational laws and regulations, while municipalities are responsible for financing and delivering compulsory and upper secondary education.

### 2.2 Earlier Grades and Contemporary School Reforms

In the early 2000s, Swedish students' academic performance declined, as evidenced by international assessments such as the Program for International Student Assessment and the Trends in International Mathematics and Science Study (The Public Health Agency of Sweden 2018). During the same period, the proportion of students meeting the requirements to progress from compulsory to upper secondary school also fell (The Swedish Government 2009). In response to these developments, the Swedish government launched a series of reforms aimed at improving educational outcomes.

In the fall of 2011, new national curricula were introduced for both compulsory (Lgr11) and upper secondary (Gy2011) education, alongside a revised and more detailed grading scale. A year later, in the fall of 2012, another key reform was implemented: grades were introduced 2 years earlier in compulsory school, shifting from year 8 to year 6 (The Swedish National Agency for Education 2011). This change in grading timing serves as the policy variation in our study, allowing us to estimate the causal effect of earlier grading on adolescent mental health. However, because the curriculum and grading scale reforms affected adjacent cohorts, they are also relevant to our empirical setup and are accounted for in our analysis.

The Lgr11 and Gy2011 curricula aimed to clarify subject‐specific syllabi and learning outcomes to make academic expectations more transparent. At the same time, the grading system was restructured. Previously, upper‐secondary schools used a four‐level scale (fail, pass, pass with distinction, pass with special distinction) while compulsory schools employed a three‐level scale (pass, pass with distinction, pass with special distinction). These were replaced with a unified six‐step scale, where grades A–E indicate passing levels and F signifies failure to meet the minimum requirements for an E, introducing a formal failing grade (F) at the compulsory school level. Although the changes were intended to encourage student motivation and ease the transition between compulsory school and upper‐secondary school, they also raised concerns about stress and potential labeling effects. Recent research by Collins and Lundstedt (2024) shows that students exposed to the revised grading scale were less likely to graduate from high school and more likely to report sleeping problems compared to peers assessed under the previous system.

Although the impact of the new curricula has yet to be formally evaluated, these reforms may have affected both academic and health outcomes across cohorts, and we take this into account in our empirical design.

The earlier grading reform also had implications for school resources. According to the Swedish National Agency for Education (The Swedish National Agency for Education 2017), over half of schools reported increased costs associated with implementing the reform, mainly due to staff training and administrative work. While such costs were meant to be reimbursed by the state, many municipalities reported that compensation was insufficient. Resource constraints, such as reduced access to student health services, lower teacher availability, or cuts in learning support, may have affected student well‐being. Although we do not have detailed data on school‐level resources, existing reports indicate a decline in the share of students receiving specialized support in the years following the reform (The Swedish National Agency for Education 2017). We explore these potential resource effects as part of our robustness checks.

### 2.3 Theoretical Context and Hypothesis: Earlier Grades and Mental Illness

For earlier grading to influence mental health, both the timing and the nature of academic feedback must matter. Human Capital Development Theory (Cunha and Heckman 2007), which incorporates insights from psychology, posits that the impact of investment and feedback on development is age‐dependent. This perspective is supported by psychological research indicating that adolescence is a critical period for brain development and heightened sensitivity to environmental factors (Fuhrmann et al. 2015). Broader theories of cognitive and social development similarly emphasize that appropriate and timely feedback is essential for both emotional and academic growth (Hattie and Timperley 2007; Rudolph et al. 2001).

While feedback is generally intended to support learning and motivation, detailed performance evaluations at an early age may have unintended negative effects, particularly for lower‐achieving students. The Cognitive Behavioral Model suggests that negative academic feedback can trigger automatic thoughts such as “I am not smart” or “I will always fail,” which are linked to internalizing disorders like depression and anxiety (Kendall and Hollon 2013). Similarly, Dweck (2006) emphasizes that how children interpret failure‐related feedback depends on their mindset and developmental stage. For some students, repeated negative feedback can result in “learned helplessness,” a state of perceived powerlessness known to predict depression (Maier and Seligman 1976; Abramson et al. 1978). In line with these theories, prior studies have documented associations between performance feedback and increased symptoms of depression and anxiety among school‐aged children (Putwain and Symes 2011; Rudolph et al. 2001).

The primary policy rationale for introducing earlier grades in Sweden was to help teachers identify students needing additional support to meet learning goals (The Swedish National Agency for Education 2017). While educators reported that the reform improved identification of struggling students, there is no evidence that it led to an actual increase in targeted support (The Swedish National Agency for Education 2017). If younger students became more aware of their academic shortcomings without receiving adequate help, this may have heightened school‐related stress, reduced motivation, and contributed to negative self‐perceptions and an increased risk of mental health problems, especially among lower‐performing students.

Another intended purpose of the reform was to enhance parental involvement by giving families earlier information about academic performance. However, this too could inadvertently heighten stress. Children's self‐image is strongly influenced by how they are perceived by key figures such as parents, teachers, and peers (Gustafsson et al. 2010). Increased scrutiny or pressure from parents based on early grading results may compound stress, particularly among low‐achieving students or late academic bloomers, for whom the gap between ability and expectations may be more pronounced. Earlier grading may thus amplify mental health risks, especially if some children would otherwise have caught up academically over time.

Based on these theoretical perspectives, we hypothesize that introducing grades at a younger age may elevate school‐related stress and thereby increase the risk of developing internalizing mental disorders such as depression and anxiety, as well as substance use disorders, which commonly emerge during adolescence (The National Board on Health and Welfare 2020).

## 3 Data

We use data from the Swedish Interdisciplinary Panel (SIP), administered by the Center for Economic Demography at Lund University. SIP covers the entire Swedish population born between 1973 and 2016 and contains linked administrative records from Statistics Sweden and the National Board of Health and Welfare.

Our study population consists of individuals born between July 1992 and June 2000, identified in the Register of the Total Population and linked to their biological or adoptive parents via the Multigenerational Register. We restrict the sample to children who continuously resided in Sweden from grade 5 through grade 9, resulting in a final sample of 524,093 individuals. Descriptive statistics are presented in Table 1.

**TABLE 1:** Descriptive statistics—mental disorder outcomes and baseline characteristics in reform and control cohorts.

	(1)	(2)	(3)	(4)
	Girls		Boys	
	Reform cohorts	Control cohorts	Reform cohorts	Control cohorts
Internalizing disorder (any)	0.0159	0.0141	0.0063	0.0057
	(0.125)	(0.118)	(0.0789)	(0.0755)
Depression	0.0082	0.0073	0.0028	0.0026
	(0.0900)	(0.0851)	(0.0525)	(0.0505)
Anxiety	0.0094	0.0071	0.0036	0.0031
	(0.0966)	(0.0843)	(0.0597)	(0.0553)
Stress	0.0021	0.0025	0.0008	0.0008
	(0.0459)	(0.0495)	(0.0285)	(0.0284)
Substance use disorder (any)	0.0019	0.0043	0.0017	0.0036
	(0.0432)	(0.0652)	(0.0407)	(0.0600)
Alcohol	0.0012	0.0036	0.0008	0.0028
	(0.0343)	(0.0602)	(0.0289)	(0.0531)
Narcotics	0.0008	0.0008	0.0009	0.0009
	(0.0289)	(0.0282)	(0.0301)	(0.0305)
Parents income (SEK)	267,093	228,636	265,131	228,988
	(288.2)	(232.2)	(246.7)	(271.4)
Low educated parents	0.0412	0.0408	0.0414	0.0405
	(0.199)	(0.198)	(0.199)	(0.197)
Moderately educated parent	0.4859	0.5287	0.4892	0.5304
	(0.500)	(0.499)	(0.500)	(0.499)
High educated parent	0.4730	0.4304	0.4695	0.4290
	(0.499)	(0.495)	(0.499)	(0.495)
Foreign‐born	0.0876	0.0712	0.0880	0.0682
	(0.283)	(0.257)	(0.283)	(0.252)
Foreign‐born parents	0.1091	0.0940	0.1066	0.0958
	(0.312)	(0.292)	(0.309)	(0.294)
Observations	91,958	162,943	98,076	171,116

*Note:* This table reports the mean probabilities and standard deviations (in parentheses) of the predefined mental disorder diagnoses and selected background characteristics, separately for girls and boys in the reform and control cohorts. The reform cohort includes all children born in Sweden within 6 months before and after January 1st in 1999 and 2000. The control cohort includes children born within 6 months before and after January 1st in 1993, 1994, and 1998 (see Section 3 for details on cohort definitions and sample construction).

We merge sociodemographic and clinical data to construct outcome variables and relevant covariates. Our main outcomes are *internalizing disorders* and *substance use disorders*, defined as binary indicators of whether the individual received a diagnosis in inpatient or specialized outpatient care during the calendar year they entered the final grade (grade 9) of compulsory school.

Internalizing disorders are emotional or behavioral conditions that are internalized by the individual, typically characterized by altered mood or affect. We define internalizing disorders to include depressive episodes and recurrent depression (ICD‐10: F32–F33); phobic anxiety disorders, other anxiety disorders, and obsessive‐compulsive disorders (F40–F42); and reactions to severe stress and adjustment disorders (F43). These conditions are also analyzed separately and hereafter referred to as *depression*, *anxiety*, and *stress*.

Substance use disorders include diagnoses related to acute intoxication, harmful use, dependence, or other mental and behavioral consequences from psychoactive substance use. We include disorders due to alcohol (F10) and other substances (F11–F16, F18–F19). These outcomes are hereafter referred to as *alcohol* and *narcotics*.

We exclude disorders unlikely to be affected by earlier academic evaluation, including organic mental disorders (F00–F09), schizophrenia and delusional disorders (F20–F29), adult personality disorders (F60–F69), and intellectual disabilities (F70–F79). Neurodevelopmental and behavioral disorders, such as autism spectrum disorders, attention‐deficit/hyperactivity disorder (ADD/ADHD), and conduct disorders, are also excluded from the main analysis but explored in robustness checks. Any observed associations with these diagnoses would likely reflect institutional or diagnostic factors rather than stress‐related mechanisms.

Migration background is captured using two indicators: (1) whether the child is foreign‐born, and (2) whether the child has foreign‐born parents (second‐generation immigrants). Parental education is categorized into three levels: (1) low education if both parents completed only compulsory schooling; (2) medium education if at least one parent completed upper secondary schooling but neither completed tertiary education; and (3) high education if at least one parent completed tertiary education. Household income is measured in the year the child turned 10 years old, prior to exposure to the reform, and is equivalized based on household composition.

## 4 Empirical Strategy

### 4.1 Identification Setup

The reform introducing earlier grades was implemented in the fall of 2012. As a result, students entering sixth and seventh grade in that school year received grades for the first time. Specifically, children born in 1998 received their first grades in eighth grade under the old system; those born in 1999 received their first grades in seventh grade; and those born in 2000 began receiving grades in sixth grade. This creates a situation in which the timing of exposure to grading is determined by birthdate relative to the school‐entry cutoff at the end of the calendar year. We exploit this cutoff to estimate the effect of earlier grading on adolescent mental health.

**FIGURE 1:**
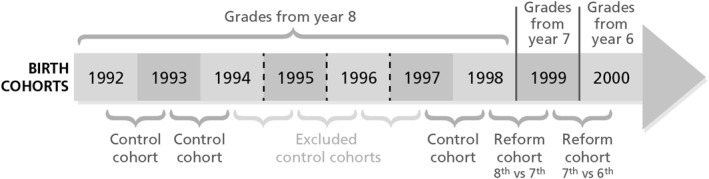
Grading system by birth cohort. This figure illustrates the grading structure across birth cohorts. Birth cohorts represent all children born between January 1st and December 31st in a given year. Re‐centered cohorts (below arrow) refer to children born between July 1st of 1 year and June 30^th^ of the next.

Our identification strategy is based on grouping individuals into re‐centered cohorts around the school‐entry cutoff. We define two reform cohorts. The first includes children born 6 months before and after January 1st, 1999. Children born before the cutoff received grades from eighth grade, while those born after received grades from seventh grade. The second reform cohort includes children born 6 months before and after January 1st, 2000, comparing those graded from seventh versus sixth grade (see Figure 1).

To isolate the impact of the grading reform, we include a set of pre‐reform control cohorts that accounts for potential discontinuities in mental health outcomes associated with school‐starting age. Specifically, we use cohorts born around January 1st in 1993, 1994, and 1998 as controls.

In contrast, we exclude cohorts born around January 1st in 1995, 1996, and 1997, since they were exposed to other concurrent educational reforms. The Gy2011 reform for upper secondary school applied to all students who started upper secondary school in the fall of 2011—that is, students born on or after January 1st, 1995. The Lgr11 reform for compulsory school was introduced at the same time, also in the fall of 2011, but it applied to students born on or after January 1st, 1996. However, students who entered ninth grade that year had already received grades under the previous system in eighth grade, and thus the grading scale did not change for them. Therefore, the grading scale reform in compulsory school primarily affected students entering eighth grade that year those born on or after January 1st, 1997.

Because policy reforms are often bundled, identifying the effect of a single reform can be challenging. We have carefully excluded cohorts likely to have been affected by other major policy changes. Nonetheless, we cannot fully rule out the influence of unobserved or delayed policy effects on the included control cohorts. We address these potential concerns in our robustness checks.

### 4.2 Difference‐In‐Discontinuities Framework

Our empirical strategy is based on a Difference‐in‐Discontinuities design, which extends the standard Regression Discontinuity (RD) framework. We estimate the effect of earlier grade introduction on adolescent mental health diagnoses by instrumenting reform exposure using month and year of birth, while controlling for age‐related and school‐starting‐age effects across the cutoff.

We begin within the RD framework, restricting the sample to the two reform cohorts—children born within 6 months before or after January 1st in 1999 and 2000. Since not all students are assigned to school cohorts strictly by calendar year due to early or delayed enrollment, we adopt a fuzzy RD design to address imperfect compliance.

Equations (1) and (2) specify a two‐stage least squares (2SLS) model:

Let *x*_*i*_ be a vector of polynomials in age (measured by birth month and centered around January 1st) for individual *i*. Treatment assignment $z_{i}$ is a binary indicator equal to one if the individual was born in January–June, and 0 otherwise.

(1) $$s_{i}=\alpha_{0}+\pi_{FS}z_{i}+x_{i}^{\prime }\alpha_{1}+{\left(z_{i}*x_{i}\right)}^{\prime }\alpha_{2}+\varepsilon_{i}$$

$$z_{i}=I\;\left[x_{i}\geq 0\right]$$

(2) $$y_{i}=b_{0}+\pi_{2SLS}{\hat{s}}_{i}+x_{i}^{\prime }b_{1}+{\left({\hat{s}}_{i}*x_{i}\right)}^{\prime }b_{2}+$$

Equation (1) models actual exposure to the reform $s_{i}$ as a function of treatment assignment $z_{i}$. The fitted value $\hat{s_{i}}$ from the first stage is then used in Equation (2), the second stage, to estimate the causal effect of the reform $\pi_{2SLS}$ on mental health outcomes *y*_*i*_.

Under the following assumptions, the fuzzy RD estimator $\pi_{2SLS}$ identifies the Local Average Treatment Effect (LATE) of earlier exposure to grading on the probability of receiving a mental disorder diagnosis among children near the cut‐off:

- *Existence of a first stage*: The instrument (birth month and year) strongly predicts exposure to the reform.
- *Independence*: Given the vector x_i_ contains a suitable set of polynomials of the running variable, then the observed and estimated counterfactual mean outcomes are jointly independent of treatment z given x=x.
- *Exclusion restriction*: The instrument affects the outcome (mental disorders) only through its impact on the reform (earlier grades).

**FIGURE 2:**
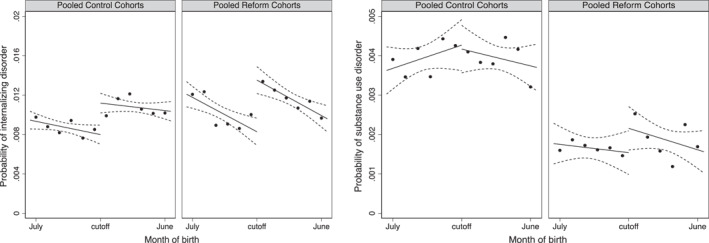
Discontinuity graphs—probability of mental disorder diagnosis by month of birth. This figure shows the mean probability of internalizing disorder and substance use disorder by month of birth, with fitted linear predictions and 95% confidence intervals clustered by birth month and year (discontinuity graphs for the separate conditions can be found in the Supporting Information S1: Appendix, Figure A2). Birth months are plotted from July to June. Estimates are presented separately for the pooled control cohorts (born around January 1st in 1993, 1994, and 1998) and the pooled reform cohorts (born around January 1st in 1999 and 2000). Discontinuities at the cutoff for control cohorts reflect age‐related effects, while discontinuities for the reform cohorts capture both age effects and the potential impact of earlier grading on mental disorder diagnoses. For cohort definitions and outcome measures, see Section 3.

First‐stage estimates and the joint significance *F*‐test (see Supporting Information S1: Appendix Table A1) confirm that the instrument strongly predicts exposure to the reform. Figure 2 displays unadjusted, reduced‐form probabilities of internalizing and substance use disorders by birth month, separately for the reform cohorts (children born around January 1st in 1999 and 2000) and control cohorts (born around January 1st in 1993, 1994, and 1998; see Section 2 for cohort definitions). For the reform cohorts, clear discontinuities in the probability of internalizing disorder diagnoses appear at the school entry cutoff, suggesting that children born just after January 1st—those exposed to earlier grading—had a higher likelihood of being diagnosed than peers born just before the cutoff. However, discontinuities are also present among the pre‐reform cohorts, implying that the observed patterns are not exclusively attributable to the grading reform. This suggests a likely violation of the independence assumption, as birth month appears correlated with mental health outcomes even in the absence of treatment.

At school entry and first exposure to grades, children born in January are nearly a year older than those born in December. This age gap affects academic performance through school‐starting‐age effects (Fredriksson and Öckert 2014) and influences the likelihood of receiving a mental disorder diagnosis, which increases with age. As a result, birth month is systematically related to both schooling and mental health outcomes, even in the absence of reform exposure.

To account for these age‐driven discontinuities, we extend the fuzzy RD model into a *Difference‐in‐Discontinuities* design (Grembi et al. 2016; Collins and Lundstedt 2024). This approach uses pre‐reform cohorts to estimate and subtract out the age‐related discontinuity in diagnosis rates, isolating the causal effect of early grading exposure.

Equations (3) and (4) specify the Difference‐in‐Discontinuities model:

(3) $$s_{i}=\gamma_{0}+\gamma_{1}z_{i}+x_{i}^{\prime }\gamma_{2}+{\left(z_{i}*x_{i}\right)}^{\prime }\gamma_{3}+R_{i}*\left\{\rho_{FS}z_{i}+x_{i}^{\prime }\gamma_{4}+{\left(z_{i}*x_{i}\right)}^{\prime }\gamma_{5}\right\}+\lambda_{Ci}+\mu_{i}$$

(4) $$y_{i}=\delta_{0}+\delta_{1}{\hat{s}}_{i}+x_{i}^{\prime }\delta_{2}+{\left({\hat{s}}_{i}*x_{i}\right)}^{\prime }\delta_{3}+R_{i}*\left\{\rho_{2SLS}{\hat{s}}_{i}+x_{i}^{\prime }\delta_{4}+{\left({\hat{s}}_{i}*x_{i}\right)}^{\prime }\delta_{5}\right\}+\lambda_{Ci}+\epsilon_{i}$$

$$R_{i}=I\;\left[\text{born}\;\text{around}\;\text{January}\;1\text{st}\;\text{in}\;1999\;\text{or}\;2000\right]$$

Variables *y*_*i*_, *z*_*i*_, and *x*_*i*_ are as defined for Equations (1) and (2). *R*_*i*_ indicates whether an individual belongs to one of the two reform cohorts, and *λ*_*Ci*_ captures birth cohort fixed effects. In this extended set‐up, ${\hat{s}}_{i}$ indicates individuals born in January–June and belonging to their expected school cohort (i.e., without early or delayed school entry) which, only for the reform cohorts, means actual exposure to the reform.

The treatment effect of the earlier grading reform is captured by $\rho_{2SLS}$ in equation ((1), (2)). To assess whether the effect varies with the timing of grading exposure, we also estimate the effects separately for each reform cohort—one exposed to earlier grading in seventh grade, and another in sixth grade. In each case, *R*_*i*_ equals 1 only for the relevant reform cohort, with the other excluded from the sample.

Estimation of the Difference‐in‐Discontinuities model relies on an additional assumption:

IV. *Constant discontinuities*: Let $f\left(x_{i}\right)$ represent the functional form of birth month, and $\delta_{1}$ denote a time‐invariant discontinuity effect. Then the counterfactual mean outcome in the absence of treatment is given by $E\left[Y_{0}|f\left(x_{i}\right)\right]=\delta_{0}+\delta_{1}.$

This assumption requires that the age‐related discontinuity in the outcome remains constant over time and is additively separable from the treatment effect. Under this condition, $\rho_{2SLS}$ identifies the change in the LATE before and after the reform, for individuals born near the school entry cutoff.

**FIGURE 3:**
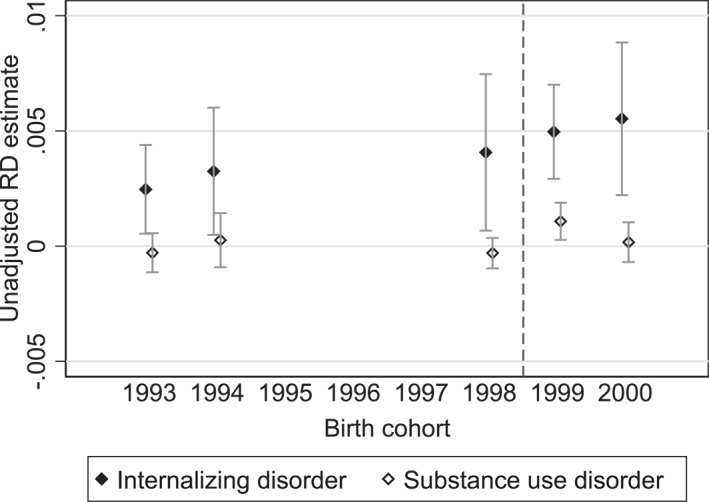
Discontinuity visualization—reduced‐form estimates of being born January–June. This figure presents reduced‐form regression discontinuity (RD) estimates of being born in January–June (vs. July–December) on the probability of receiving a mental disorder diagnosis in the calendar year the student enters grade 9 (the final grade of compulsory school). Estimates are plotted separately by birth cohort and reflect the discontinuity in mental health outcomes at the school entry cutoff. Spikes represent 95% confidence intervals clustered by birth month and year. The sample includes all children born in Sweden within 6 months before and after January 1st in 1993, 1994, 1998, 1999, and 2000. For cohort definitions and outcome measures, see Section 3.

One concern is that this assumption may not hold. To assess if the discontinuities are constant, in Figure 3, we present reduced‐form RD estimates of being born in January–June versus July–December across all relevant birth cohorts. The results suggest an upward trend over time in the difference in internalizing disorders around the cutoff. To address this, we extend our model to include a linear cohort‐trend, assuming a growth rate in the age effect. This is our preferred specification. In the robustness section, we assess the sensitivity of our results to this assumption by re‐estimating the model without cohort‐specific time trends.

A further threat to identification is a possible violation of the exclusion restriction, as we cannot fully rule out the influence of other contemporaneous reforms or delayed effects from the *Lgr11* curriculum reform. To mitigate this, we exclude cohorts directly exposed to either the new curricula or the grading scale adjustments. Additionally, we conduct a placebo test using the 1998 control cohort—the first cohort to follow the Lgr11 implementation but not exposed to earlier grading. This allows us to assess whether the observed effects are plausibly driven by earlier grading, rather than other policy changes.

Standard errors are clustered at the birth month and year level. Given the discrete nature of our running variable (birth month), we also report robust standard errors in the robustness section, as these may provide improved coverage properties (Kolesár and Rothe 2018). Furthermore, we test the sensitivity of our results to model specification by estimating second‐order polynomials in the running variable and evaluating alternative bandwidths on either side of the cutoff.

## 5 Results

### 5.1 The Effect of Earlier Grading on Mental Disorders by the End of Compulsory School

Our main results, presented in Table 2, show that exposure to 1 year earlier grading (i.e., receiving grades in sixth vs. seventh grade, or in seventh vs. eighth grade) is significantly associated with a 0.31% point increase in the probability of being diagnosed for internalizing disorders in the year the student enters the final year of compulsory school. We also find that earlier grading is associated with a 0.10% point increase in the likelihood of a substance use disorder diagnosis; this estimate is only weakly statistically significant.

**TABLE 2:** Main results—the effect of earlier grading on mental disorder diagnoses at the end of compulsory school.

	Internalizing disorder				Substance use disorder		
	Any	Depression	Anxiety	Stress	Any	Alcohol	Narcotics
	(1)	(1a)	(1b)	(1c)	(2)	(2a)	(2b)
Full sample							
Earlier grades reform	0.0031**	0.0022**	0.0013	0.0003	0.0010*	0.0017***	−0.0006
	(0.0015)	(0.0010)	(0.0012)	(0.0005)	(0.0006)	(0.0005)	(0.0004)
Baseline	0.0098	0.0049	0.0051	0.0016	0.0039	0.0032	0.0009
Observations: 524,093							
Girls							
Earlier grades reform	0.0057**	0.0039**	0.0023	0.0005	0.0009	0.0019**	−0.0008
	(0.0025)	(0.0018)	(0.0019)	(0.0007)	(0.0011)	(0.0008)	(0.0007)
Baseline	0.0141	0.0073	0.0071	0.0025	0.0043	0.0036	0.0008
Observations: 254,901							
Boys							
Earlier grades reform	0.0006	0.0005	0.0003	0.0000	0.0011	0.0015*	−0.0003
	(0.0018)	(0.0010)	(0.0012)	(0.0006)	(0.0009)	(0.0008)	(0.0005)
Baseline	0.0057	0.0026	0.0031	0.0008	0.0036	0.0028	0.0009
Observations: 269,192							

*Note:* This table presents the estimated effects of earlier grading (in grade 6 vs. grade 7 and grade 7 vs. grade 8) on the probability of receiving a mental disorder diagnosis during the calendar year the student enters grade 9 (the final grade of compulsory school). Each column and subgroup is estimated using a separate Difference‐in‐Discontinuities regression. The reported coefficients (*ρ*_2SLS_) represent the Local Average Treatment Effect of exposure to 1 year earlier grading. The sample includes all children born in Sweden within 6 months before and after January 1st in 1993, 1994, 1998, 1999, and 2000. The “Baseline” row shows the mean diagnosis rate in the pooled control cohorts. Robust standard errors clustered by birth month and year are reported in parentheses. For cohort definitions and outcome measures, see Section 3.

^*^ *p* < 0.1.

^**^ *p* < 0.05.

^***^ *p* < 0.01.

While the absolute effects may appear small, they are substantial relative to the low baseline rates of diagnosis at this age. The mean probability of being diagnosed with an internalizing disorder is 0.98%, and 0.39% for substance use disorders. Accordingly, the observed effects correspond to a 32% increase in diagnoses for internalizing disorders and a 26% increase for substance use disorders.

When disaggregating by sex, the effect on internalizing disorders is concentrated among girls. Exposure to earlier grading increases the probability of diagnosis among girls by 0.57% points, corresponding to a 40% relative increase. Among boys, the estimated effect is small and not statistically significant. For substance use disorders, the estimated effects are similar in magnitude for both sexes—0.09% points for girls and 0.11% points for boys—but neither is statistically significant.

Further analysis of subcategories within internalizing disorders reveals that the overall effect is primarily driven by depression among girls. The earlier grading reform is associated with a 0.39% point increase in the likelihood of a depression diagnosis among girls, equivalent to a 53% increase. The estimated effect on anxiety is also positive (0.23% points, or a 32% increase), though not statistically significant. For stress‐related diagnoses, the effect is smaller and likewise not statistically significant.

Our findings indicate that the observed increase in substance use disorders is primarily driven by diagnoses related to alcohol use. One‐year earlier grading is significantly associated with 0.17% points increase in the probability of alcohol‐related diagnosis in the full sample, relating to a relative effect by around 53%. The effect on alcohol appears to be quite similar between the sexes, 0.19% points among girls and 0.15% points among boys (weakly significant), which relates to 53–54% increase in the probability of alcohol diagnosis. In contrast, the estimated effects for narcotics‐related diagnoses are small, negative, and not statistically significant. Given the varying effects between boys and girls, all subsequent analyses are separated by sex.

To examine whether these effects reflect broader patterns or are confined to individuals near the school‐starting‐age cutoff, we consider the discontinuity graphs of the raw data presented in Figure 2. For internalizing disorders, the pre‐ and post‐cutoff trends appear parallel, suggesting that the estimated effects may generalize beyond those close to the cutoff. By contrast, for substance use disorders, the discontinuity appears more localized, implying a weaker case for generalizability. We consider the generalizability of our findings further in the robustness section.

### 5.2 Effects of Earlier Grading in Sixth or Seventh Grade

In our baseline model, we estimated the average effect of receiving grades 1 year earlier—either in sixth (instead of seventh) or seventh (instead of eighth) grade. To assess whether the overall effect is primarily driven by one of these transitions, we now estimate the effects separately for the two reform cohorts: those first graded in sixth grade and those first graded in seventh grade.

When interpreting the effects of earlier grading in sixth grade, it is important to consider that the youngest students in the seventh grade reform cohort provide a relevant comparison group. Our earlier analyses indicate that the impact of earlier grading on internalizing disorders is relatively stable across birth months within a cohort, suggesting a potentially additive treatment effect. If so, the estimated impact of sixth grade grading should be interpreted as incremental to that of seventh grade grading. However, we find no evidence of cumulative effects for substance use disorders.

**FIGURE 4:**
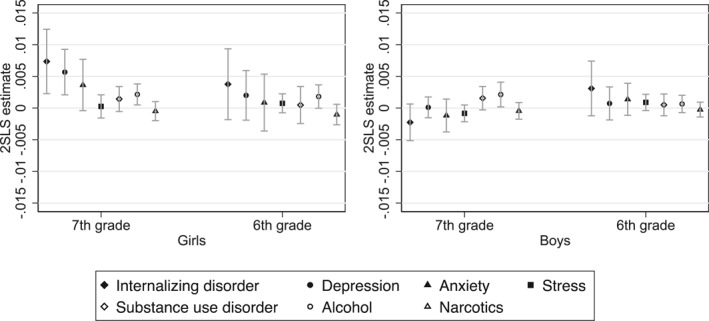
The effect of earlier grading in sixth or seventh school year on mental disorder diagnoses. This figure presents the estimated effect of earlier exposure to grading on the probability of receiving a mental disorder diagnosis in the calendar year the student enters grade 9 (the final grade of compulsory school). Spikes represent 95% confidence intervals clustered by birth month and year. Each point displays the 2SLS treatment effect estimate (*ρ*_2SLS_) from separate Difference‐in‐Discontinuities regressions. Estimates for earlier grading in grade 7 (rather than grade 8) are based on children born 6 months before and after January 1st, 1999; estimates for earlier grading in grade 6 (rather than grade 7) are based on the cohort born around January 1st, 2000. Control cohorts include those born around January 1st in 1993, 1994, and 1998. For cohort definitions and outcome measures, see Section 3.

Figure 4 presents the estimated effects of earlier grading in sixth and seventh grade. The results indicate that the observed increase in internalizing disorders is primarily driven by exposure to earlier grading in seventh grade. Consistent with our main findings, these effects are concentrated among girls and are strongest for depression, followed by anxiety. In contrast, the estimated effects of earlier grading in sixth grade on internalizing disorders are smaller in magnitude and not statistically significant for either girls or boys.

The rise in substance use disorders observed in the main results also appears to be driven predominantly by the seventh grade reform cohort, for both genders, although the estimates are not statistically significant. Further disaggregation by diagnosis type reveals that the increase is attributable to alcohol‐related disorders. Among girls, this effect is linked to earlier grading in both sixth and seventh grade, while among boys, it is associated only with earlier grading in seventh grade.

### 5.3 Timing of the Effect of Earlier Grading

Our main results estimate the impact of earlier grading on mental health outcomes during the calendar year students in which students enter grade 9, the final year of compulsory schooling. Before the reform, mental health outcomes measured in grade 9 corresponded to a 1‐year follow‐up after initial exposure to grading. However, this approach risks overlooking potential dynamic effects, as the consequences of earlier grading may not be most pronounced at that specific point in time. It is plausible that the reform triggered anticipatory stress in earlier school years or, alternatively, thay it had an immediate but short‐lived impact that had already dissipated by grade 9.

**FIGURE 5:**
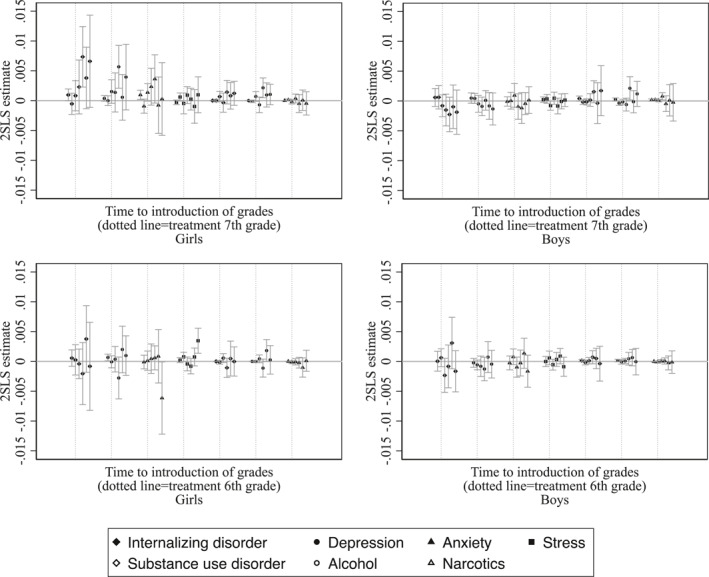
Timing of the effect of earlier grading on mental disorder diagnoses. This figure presents the estimated effect of earlier grading on the probability of receiving a mental disorder diagnosis from the year the student enters grade 5 through the second year of upper secondary school. The final follow‐up year (second year of upper secondary) is only available for the cohort exposed to earlier grading in grade 7 (vs. grade 8). Each point represents a 2SLS treatment effect estimate (*ρ*_2SLS_) from separate Difference‐in‐Discontinuities regressions. Estimates are presented separately for the two reform cohorts: one exposed to earlier grading in grade 7 and the other in grade 6. Spikes represent 95% confidence intervals clustered by birth month and year. The sample includes children born in Sweden within 6 months before and after January 1st in 1999 (for seventh vs. eighth grade) or 2000 (for sixth vs. seventh grade), along with control cohorts born around January 1st in 1993, 1994, and 1998 (for cohort definitions and outcome measures, see Section 3).

To examine the timing of these effects more closely, we estimate the impact of earlier grading on mental disorders from the year students enter grade 5 (1–2 years prior to exposure) through to second year of upper‐secondary school (up to 6 years after). This period captures the remainder of compulsory schooling and the first 2 years of upper secondary education, which most students enter directly after grade 9. To better understand the underlying mechanisms, we conduct this analysis separately for the two reform cohorts: one exposed to earlier grading in grade 7 (compared to grade 8), which appears to be the primary driver of the increase in mental disorders, and the other in grade 6 (compared to grade 7). The results are presented in Figure 5.

The findings suggest that for girls exposed to earlier grading in grade 7, the elevated probability of being diagnosed for internalizing disorders begins to emerge before grade 9, particularly for anxiety‐related conditions, although depression is also somewhat elevated. This indicates an immediate mental health response to the reform. The effect appears to accumulate over time, peaking in grade 9, before declining as students transition into upper secondary school. Notably, the effect rises again in the following year (the final follow‐up year), although this is observed only for depression.

Among boys, the effects of earlier grading on internalizing disorders remain small throughout, consistent with our main results. If anything, the estimates suggest a modest protective effect, although these results are not statistically robust and should be interpreted with caution.

For substance use disorders, the increase in diagnoses observed in grade 9, driven by alcohol‐related disorders, does not appear to be preceded by elevated diagnosis rates in earlier years.

## 6 Heterogeneity Analysis

In this section, we explore whether the effects of earlier grading on mental disorders vary by migration background, parental socioeconomic status (income and education), and academic performance as measured in quartiles of the final grade point average (GPA) distribution at the end of compulsory school.

**FIGURE 6:**
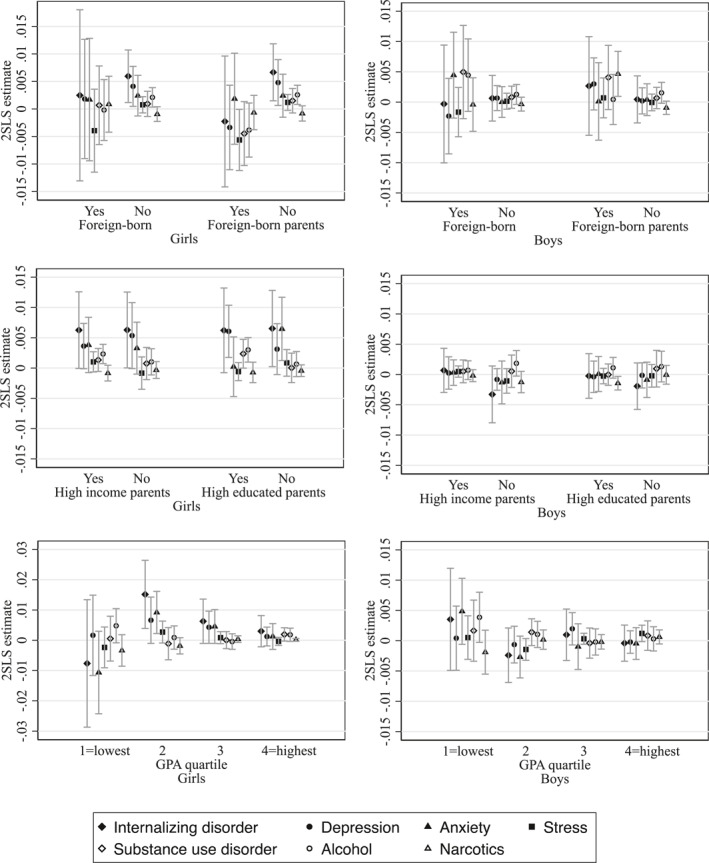
Heterogeneity analysis—the effect of earlier grading by background characteristics and GPA quartiles. This figure shows the estimated effect of earlier grading on the probability of receiving a mental disorder diagnosis in the calendar year the student enters the final grade (grade 9) of compulsory school. Each point represents the 2SLS treatment effect estimate (*ρ*_2SLS_) from separate difference‐in‐discontinuities regressions, stratified by migration background (foreign‐born or not, foreign‐born parents or not), parental socioeconomic status (above or below median income; at least one tertiary‐educated parent or not), and student academic performance (GPA quartiles). Spikes represent 95% confidence intervals clustered by birth month and year. The sample includes all children born in Sweden within 6 months before and after January 1st in 1993, 1994, 1998, 1999, and 2000 (for cohort definitions and outcome measures, see Section 3).

The results, presented in Figure 6, indicate some variation across subgroups, though most differences are not statistically significant. Among girls, the increase in mental disorders following earlier grading is concentrated among those who are Swedish‐born with Swedish‐born parents. This pattern is evident for both depression and alcohol‐related diagnoses. In contrast, having foreign‐born parents appears somewhat protective against alcohol‐related disorders, although the negative estimates are not statistically distinguishable from zero. Among boys, a foreign background appears to be linked to a higher probability of receiving a substance use disorder diagnosis after exposure to earlier grading. Specifically, alcohol‐related diagnoses increase among foreign‐born boys, while narcotics‐related diagnoses increase among boys with foreign‐born parents. It is important to note that individuals with a foreign background constitute a smaller share of the sample, which may explain the wider confidence intervals and less stable estimates observed in these subgroups.

We observe limited heterogeneity by parental education and income. Among girls, the effect of earlier grading on alcohol‐related diagnoses is evident only for those with highly educated or above‐median‐income parents, though these differences are not statistically significant. The effect on depression is observed among girls with highly educated parents, while the effect on anxiety is present only among girls with low‐educated parents. Among boys, results are largely consistent with the baseline findings—there is no significant effect of earlier grading on internalizing disorders in any subgroup. However, there are indications that the effect on alcohol‐related diagnoses is higher among boys from low‐income households.

The heterogeneity analysis by academic performance suggests that the effect of earlier grading on internalizing disorders among girls is primarily driven by those in the second GPA quartile (relatively low‐performing), with some indication of effects among girls in the third quartile (moderately performing), although the latter is not statistically significant. No effect is observed among girls in the top GPA quartile, and if anything, a non‐significant protective effect on anxiety is found among those in the bottom quartile. In contrast, the probability of alcohol‐related diagnoses is elevated among girls in both the bottom and top GPA quartiles, though these estimates are not statistically significant. Among boys, earlier grading does not significantly affect internalizing disorders in any GPA quartile. However, anxiety appears somewhat higher in the bottom quartile and slightly lower in the second quartile. The increase in alcohol‐related diagnoses also appears concentrated among boys in the bottom performance quartile.

## 7 Robustness

### 7.1 Alternative Model Specifications

We evaluate the sensitivity of our findings by: (1) comparing robust versus clustered standard errors, (2) removing the linear cohort trend in the school‐starting‐age effect, (3) using only the cohort re‐centered around January 1st, 1998 as a control group, (4) applying narrower bandwidths around the cutoff in the RD model, and (5) including a second‐order polynomial in the running variable (birth month). Summary results to these alternative model specifications are presented in the Supporting Information S1: appendix Table A2, with full estimates for all outcomes presented in Supporting Information S1: Tables A3–A7.

For internalizing disorders, the estimates are stable across all specifications for the full sample and for girls. Among boys, the point estimates are more variable but consistently remain small and not statistically significant. For substance use disorders, the estimates are more sensitive to changes in the RD model specification, but the main results appear to be, if anything, conservative.

### 7.2 Test for Manipulation

We test whether the reform may have triggered manipulation of school cohort assignment, such as delaying or advancing school start dates. Given that such practices are rare in Sweden, and the reform was not announced until 2010, strategic manipulation is unlikely. Supporting Information S1: Figure A3 shows no discontinuities in birth month distributions around the cutoff, supporting the validity of the RD design.

### 7.3 Covariate Balance

Unlike standard RD designs, our Difference‐in‐Discontinuities approach does not require complete continuity in covariates at the cutoff. Instead, it relies on the assumption of *constant discontinuities*—that any discontinuities in covariates at the threshold are similar between reform and control cohorts.

Standardized means for the covariates by birth month are plotted for reform and control cohorts in Supporting Information S1: Figure A4. The discontinuity graphs reveal no visible inconsistencies at the cutoffs, with one exception: a somewhat larger discontinuity for the share of students with foreign‐born parents.

To formally test for balance, we re‐estimate Equation (4) using each predetermined characteristic as the dependent variable (see Supporting Information S1: Table A8). The results show no statistically significant associations for most covariates. A weak and inconsistent relationship emerges for having foreign‐born parents, but the effect is small and not robust across specifications.

### 7.4 Placebo‐Test

To further assess the robustness of our findings, we simulate exposure to the reform for the 1998 cohort, which was not actually affected. Supporting Information S1: Figure A5 shows no effect of this placebo exposure on any outcome, reinforcing the credibility of our identification strategy.

### 7.5 Exploring the Mechanisms

A key concern is that the observed increase in mental disorders may not reflect a true rise in underlying conditions but rather changes in diagnostic practices or school resource allocation. One may theorize that, in response to earlier grading, parents sought psychiatric evaluations more frequently, anticipating that a diagnosis could lead to additional educational support. Similarly, schools or parents may have initiated evaluations earlier to secure resources for interventions. Another concern is that resources originally intended for student support may have been diverted toward administrative costs associated with implementing earlier grading, potentially affecting student mental health indirectly.

However, Swedish education law states that a diagnosis is neither a requirement for receiving support nor a guarantee of it (The Swedish National Agency for Education 2024). Support is based on whether a student is at risk of not meeting academic goals, regardless of diagnostic status. If a diagnosis exists, its relevance to school functioning must be evaluated, but it should not serve as the sole criterion for support. This legal framework limits the potential for strategic parental behavior and reduces the likelihood that our main results are driven by changes in diagnostic incentives.

If strategic responses or shifts in diagnostic behavior were influencing our results, we would expect to see increases not only in internalizing disorders but also in other diagnoses commonly linked to school support, such as neurodevelopmental disorders. To test this, we examine whether the reform increased the probability of diagnoses for ADD/ADHD (ICD‐10 F90), conduct disorder (ICD‐10 F91), autism spectrum disorders (ICD‐10 F84), or any behavioral and emotional disorders that typically onset in childhood (ICD‐10 F90–F98).

We find no evidence that earlier grading led to increases in the probability of these diagnoses (see Supporting Information S1: Table A9 in the appendix). This suggests that the observed effects are unlikely to be driven by strategic diagnostic behavior or shifts in resource allocation.

## 8 Discussion

Our results show that exposure to earlier grading leads to an increased probability of receiving a mental disorder diagnosis by the end of compulsory school. The effects differ across disorder types—internalizing disorders (such as depression and anxiety) and substance use disorders—and between girls and boys.

For girls, earlier grading increases the likelihood of internalizing disorders, primarily depression and, to a lesser extent, anxiety. Although the absolute effect sizes are modest, the low baseline probability of diagnosis (around 1.5%) means that the reform increases the likelihood of diagnosis by approximately 40–53%. These effects are consistent across several robustness checks, including covariate balance, placebo tests, and alternative model specifications. We also find evidence suggesting that the treatment effect is generalizable beyond the oldest students in a cohort for internalizing disorders, although this does not appear to be the case for substance use disorders.

We also find a positive association between earlier grading and substance use disorders, primarily driven by alcohol‐related diagnoses. Again, the absolute effects are small, but due to the low baseline diagnosis rate (below 0.5%), the relative increase is about 53–54%. However, the effect among boys is not robust, and the results for girls are somewhat sensitive to model adjustments. We therefore interpret this finding with caution and recommend further research on the relationship between academic evaluation and substance use.

### 8.1 Gender Differences in Response to Grades

Our findings indicate that the mental health impact of earlier grading is concentrated among girls. While we are not aware of prior studies examining the effect of introducing grading on mental health at the extensive margin, several related studies suggest that girls and young women are more responsive to school reforms. Högberg et al. (2021) found that health impacts of curriculum and grading reforms in Sweden were stronger for girls. Similarly, Klapp (2015) and Sjögren (2010) found that earlier grading affected school performance and attainment more for girls. Linder et al. (2023) also report that grading leniency had protective effects on mental health, particularly among young women.

Why girls respond more strongly remains an open question. One explanation is that school performance and grades are more salient for girls during adolescence. Prior research shows that girls report more stress and pressure related to academic evaluation (West and Sweeting 2003), and that young women place greater value on grades than young men (Ahn et al. 2019), which may influence subject and career choices. Additionally, girls exhibit a significantly higher prevalence of internalizing disorders, both in clinical diagnoses and self‐reported symptoms, and for psychotropic medication (The Public Health Agency of Sweden 2018; The National Board of Health and Welfare 2017). While this likely reflects real differences in mental health, it may also be shaped by differential help‐seeking behavior or diagnostic thresholds between genders (Kovess‐Masfety et al. 2014). Similarly, differences in self‐reported health may reflect the varying tendency to report symptoms between groups.

If diagnoses among girls reflect different thresholds or symptom severity than among boys, this may help explain why we find stronger effects of earlier grading for girls. Future research should further investigate gender‐specific pathways linking academic evaluation to mental health.

### 8.2 Heterogeneity by Background Factors

Beyond gender, we find limited evidence of systematic heterogeneity based on migration background or parental socioeconomic status. While there are small variations, such as somewhat higher effects among Swedish‐born girls with Swedish‐born parents, or among girls with highly educated or higher‐income parents, these differences are not statistically significant and may reflect variation in healthcare access or utilization. Further research is needed to determine whether family background moderates the relationship between grading and mental health.

### 8.3 Heterogeneity by Academic Performance

We do find clearer evidence of heterogeneity across the academic achievement distribution. The increase in internalizing disorders—particularly depression and anxiety—is concentrated among girls in the second‐lowest GPA quartile, with effects up to four times higher than the average treatment effect. Smaller effects are also observed among girls in the second‐highest quartile. By contrast, girls in the highest and lowest GPA quartiles appear unaffected or even slightly protected against anxiety.

That high‐achieving girls are less affected is intuitive, their grades likely affirm their abilities and are reinforced by support from teachers and parents. The finding that the lowest‐achieving girls are not significantly affected, or may even benefit, requires further investigation. One possibility is that these students, who often do not receive final grades, may already have adjusted their expectations, making them less vulnerable to evaluative stress. The heterogeneity along the performance distribution underscores the need for more nuanced research into how students with different academic profiles respond to grading pressure.

### 8.4 Grading in Grade 6 Versus Grade 7

We find that earlier grading in grade 7 significantly increases the likelihood of internalizing disorders, while the effect of introducing grades in grade 6 is smaller and not statistically significant. However, interpreting the grade 6 effect is challenging due to the quasi‐experimental setup, students in this cohort followed a cohort that had already experienced earlier grading in grade 7. Thus, the impact of earlier grading in sixth grade may depend on how earlier grading in seventh grade affected the youngest students in that cohort. If the effects of grading extend beyond the oldest students in a class, as our findings suggest, then the grade 6 estimates likely reflect additional or cumulative exposure. For substance use disorders, by contrast, the effects appear localized to the oldest students in each grade cohort.

### 8.5 Limitations and Broader Implications

A key strength of this study is the use of comprehensive administrative register data, which enables detailed analysis of heterogeneity and ensures high measurement precision. However, a limitation is that our outcomes capture only diagnosed cases in inpatient and specialized outpatient care, likely representing more severe cases. Many adolescents with mental health problems do not seek or receive formal diagnoses, particularly through specialized services (Bremberg and Dalman 2015). Mental health care is also commonly provided through school and youth health services, which are not captured in our data.

Survey data suggest that between 9 and 28% of adolescent girls in Sweden report frequent feelings of depression or anxiety, and between 8 and 18% experience severe anxiety or sleep problems (Bremberg and Dalman 2015). Among boys, these rates are approximately half. Our results, therefore, likely reflect the most severe end of the mental health distribution. That we observe significant effects on this rare but high‐validity measure suggests that earlier grading could also influence a wider range of, less severe, mental health problems. Further research using more sensitive indicators, such as prescriptions or self‐reported symptoms, could help clarify this broader impact.

Finally, we are unable to assess longer‐term outcomes due to the limited sample period. While we observe smaller effects shortly after exposure to grading, the main effects appear in grade 9, consistent with a cumulative impact during a sensitive developmental period (Fuhrmann et al. 2015). The effects diminish in the first year of upper secondary school, only to increase again slightly thereafter, possibly reflecting the pressure of final grades and educational transitions. Future research should examine the longer‐term effects of earlier grading on mental health and academic performance.

## 9 Conclusion

This study finds that the introduction of earlier grading in Sweden's compulsory school system increased the likelihood of internalizing disorder diagnoses among girls, particularly depression. By isolating the effects of a specific policy reform, the results offer broader insights into whether the growing emphasis on performance monitoring in schools may be contributing to the rise in mental health problems among adolescents.

Furthermore, the findings may offer insights into why internalizing disorders are more prevalent among girls. They suggest that school‐related factors could contribute to the early development of this gender gap—a gap that tends to emerge during the school years and often persists into adulthood.

## Ethics Statement

The study is approved by the Regional Ethics Committee in Lund (2012/627) and the Swedish Ethical Review Authority (2021–02630).

## Conflicts of Interest

The authors declare no conflicts of interest.

## Supporting information

Supporting Information S1

## Data Availability

The data that support the findings of this study are available from Statistics Sweden; The National Board on Health and Welfare. Restrictions apply to the availability of these data, which were used under license for this study. Data are available from the author(s) with the permission of Statistics Sweden; The National Board on Health and Welfare.

## Endnotes

^1^
This rule is not binding, and parents may request either early or delayed school entry. As a result, a small share of children are assigned to school cohorts that do not correspond to their birth year, see Supporting Information S1: Appendix Figure A1.
