# Supplementary material for: Adolescent Mental Health: Impact of Introducing Earlier Compulsory School Grades
Source: Health Econ. 2025 Jul 9;34(9):1731–46. doi: 10.1002/hec.4982 (PMC12316578; doi:10.1002/hec.4982)
Supplement: Supplementary file 1 — Supporting Information S1 [file HEC-34-1731-s001.docx]

Adolescent mental health:

Impact of introducing earlier compulsory school grades:

Online Appendix

|  |  |
| --- | --- |

**Figure A1: Assignment to school cohort by month of birth**

Note: This figure shows the average probability of being assigned to an earlier or later school cohort. School cohort is determined by the year the child conducts national 9th grade exams and birth cohort refers to the child’s calendar-year birth cohort. "Late" assignment refers to placement with younger peers, while "early" assignment refers to placement with older peers. Probabilities are plotted in monthly bins, from July through June. The sample includes all children born in Sweden within six months before and after January 1st in the 1993, 1994, 1998, 1999, and 2000 cohorts (see Section 3 for details).

Among children born between February and October, fewer than 5 percent are placed in a non-standard cohort. In contrast, school entry deviations are more common for children born near the cutoff: approximately 6 to 10 percent of those born in November or December are assigned to a later school cohort (starting school a year later than their birth year cohort), while about 6 percent of children born in January are assigned to an earlier school cohort (starting school a year early).

|  |  |
| --- | --- |
|  |  |
|  |  |

**Figure A2 Discontinuity graphs – Probability of diagnosis by month of birth**

Note: This figure displays the mean probability of mental disorder diagnoses by month of birth (July to June), along with fitted linear predictions and 95% confidence intervals (clustered by birth month and year). Estimates are shown separately for the pooled control cohorts (children born around January 1st in 1993, 1994, and 1998) and the pooled reform cohorts (children born around January 1st in 1999 and 2000). Discontinuities at the cutoffs for the control cohorts reflect age effects by month of birth, while discontinuities at the cutoffs for the reform cohorts reflect both age effects and the potential impact of one-year earlier exposure to grades on mental disorder diagnoses.

**Table A1: First stage – the effect of birth month on exposure to earlier grading**

|  | (1) |
| --- | --- |
|  | **Earlier grades reform** |
| Born January-June*reform (earlier grades reform instrument) | 0.9321*** |
|  | (0.0079) |
| Constant | -9.1836*** |
|  | (1.6386) |
| Observations | 524,093 |
| R-squared | 0.9222 |

Note: This table presents the first-stage results for the instrument—specifically, the effect of being born in January–June during a reform year on actual exposure to earlier grading. Exposure is defined based on alignment between birth cohort and school cohort, with school cohort inferred from the year the student took national exams in grade 9. The sample includes all children born in Sweden within six months before and after January 1st in 1993, 1994, 1998, 1999, and 2000 (see Section 3 for sample details). Robust standard errors clustered by birth month and year are reported in parentheses. ***p<0.01, **p<0.05, *p<0.1

**Table A2: Robustness – alternative model specifications**

|  | **Internalizing disorder** | | |  |  | **Substance use disorder** | | |
| --- | --- | --- | --- | --- | --- | --- | --- | --- |
|  | Full sample | Girls | Boys |  |  | Full sample | Girls | Boys |
| Main results | 0.0031** | 0.0057** | 0.0006 |  |  | 0.0010* | 0.0009 | 0.0011 |
|  | (0.0015) | (0.0025) | (0.0018) |  |  | (0.0006) | (0.0011) | (0.0009) |
|  |  |  |  |  |  |  |  |  |
| Robust SE | 0.0031* | 0.0057** | 0.0006 |  |  | 0.0010 | 0.0009 | 0.0011 |
|  | (0.0017) | (0.0028) | (0.0018) |  |  | (0.0008) | (0.0012) | (0.0011) |
|  |  |  |  |  |  |  |  |  |
| Without SSA | 0.0024* | 0.0063*** | -0.0012 |  |  | 0.0008* | 0.0011 | 0.0005 |
| time trend | (0.0013) | (0.0021) | (0.0014) |  |  | (0.0005) | (0.0009) | (0.0010) |
|  |  |  |  |  |  |  |  |  |
| Only control | 0.0033 | 0.0048 | 0.0019 |  |  | 0.0014* | 0.0012 | 0.0017 |
| cohort 1998 | (0.0023) | (0.0039) | (0.0025) |  |  | (0.0008) | (0.0011) | (0.0011) |
|  |  |  |  |  |  |  |  |  |
| RD Bandwidth | 0.0029* | 0.0051* | 0.0008 |  |  | 0.0018*** | 0.0023** | 0.0013 |
| Aug to May | (0.0017) | (0.0028) | (0.0020) |  |  | (0.0006) | (0.0010) | (0.0010) |
|  |  |  |  |  |  |  |  |  |
| RD Bandwidth | 0.0024 | 0.0049 | 0.0001 |  |  | 0.0021*** | 0.0034*** | 0.0010 |
| Sep to Apr | (0.0019) | (0.0030) | (0.0019) |  |  | (0.0005) | (0.0010) | (0.0010) |
|  |  |  |  |  |  |  |  |  |
| RD Running | 0.0035* | 0.0054* | 0.0018 |  |  | 0.0030*** | 0.0045*** | 0.0014 |
| variable sq | (0.0020) | (0.0028) | (0.0022) |  |  | (0.0007) | (0.0013) | (0.0015) |

Note: This table presents the main results for internalizing disorders and substance use disorders (from Table 2) in the top rows, followed by estimates from alternative model specifications listed in the left column. These include (1) adjustments to the standard error (SE) estimation - robust (versus clustered standard errors by birth month and year as baseline), (2) removal of the linear cohort trend in school-starting-age effects, (3) using only the 1998 cohort as a control group (excluding the Jan 1^st^ 1993 and 1994 control cohorts), (4) narrower RD bandwidths, and (5) second-order polynomial specifications of the running variable (birth month). Full estimates for all outcomes are presented in Tables A3–A7. All models are estimated using the difference-in-discontinuities framework. ***p<0.01, p<0.05, p<0.1

Extended discussion of Table A.2: For internalizing disorders, the estimates are stable across all specifications for the full sample and for girls. Among boys, the point estimates are more variable but consistently remain not statistically significant. Notably, when using only the 1998 cohort as a control group, the results confirm the pattern shown in Figure 3 – there is no statistically significant difference in school-starting-age effects before and after the reform at the 95% level. This supports the assumption of stable discontinuities across cohorts. While our main results use additional control cohorts (1993 and 1994) to increase statistical power, the comparison shows that this improves precision without materially affecting the point estimates.

For substance use disorders, the estimates are less stable and more sensitive to changes in model specification, particularly when adjusting the RD bandwidth and polynomial order. While our main estimate lies at the lower bound of the sensitivity range, all alternative specifications point to an increase in substance use disorders following the grading reform, with the effect primarily driven by girls. The choice between clustered and robust standard errors has little impact on statistical significance or inference.

The consistency of the results for internalizing disorders across model specifications supports the generalizability of the treatment effect to a broader population of younger students within a school year. In contrast, the relative instability of results for substance use disorders suggests that the effects may be more localized to older cohorts within a school year and that our main estimates may underestimate the true treatment effect. This interpretation aligns with the visual patterns observed in Figure 2, which presents binned means by birth month.

**Table A3: Robust standard errors - the effect of earlier grading on mental disorder diagnoses**

|  | (1) | (2) | (3) | (4) | (5) | (6) | (7) |
| --- | --- | --- | --- | --- | --- | --- | --- |
|  | **Internalizing disorder** | **Depression** | **Anxiety** | **Stress** | **Substance use disorder** | **Alcohol** | **Narcotics** |
| **Full sample** | | | | | | | |
| Earlier grades reform | 0.0031* | 0.0022* | 0.0013 | 0.0003 | 0.0010 | 0.0017** | -0.0006 |
|  | (0.0017) | (0.0012) | (0.0013) | (0.0006) | (0.0008) | (0.0007) | (0.0005) |
| Observations: 524,093 |  |  |  |  |  |  |  |
| **Girls** | | | | | | | |
| Earlier grades reform | 0.0056** | 0.0039* | 0.0023 | 0.0005 | 0.0009 | 0.0019* | -0.0008 |
|  | (0.0028) | (0.0021) | (0.0021) | (0.0011) | (0.0012) | (0.0010) | (0.0007) |
| Observations: 254,901 |  |  |  |  |  |  |  |
| **Boys** | | | | | | | |
| Earlier grades reform | 0.0006 | 0.0005 | 0.0003 | 0.0000 | 0.0011 | 0.0015* | -0.0003 |
|  | (0.0018) | (0.0012) | (0.0013) | (0.0007) | (0.0011) | (0.0009) | (0.0007) |
| Observations: 269,192 |  |  |  |  |  |  |  |

Note: This table presents the estimated effects of earlier grading (in 6th versus 7th grade and 7th versus 8th grade) on the probability of receiving a mental disorder diagnosis in the year the student enters grade 9 of compulsory school. Each column and subgroup is estimated using a separate Difference-in-Discontinuities regression. The reported coefficients (ρ_2SLS_) represent the Local Average Treatment Effect of exposure to one year earlier grading. The sample includes all children born in Sweden within six months before and after January 1st in 1993, 1994, 1998, 1999, and 2000 (see Section 3 for sample details). Robust standard errors are reported in parentheses. ***p<0.01, **p<0.05, *p<0.1

**Table A4: No time trends - the effect of earlier grading on mental disorder diagnoses**

|  | (1) | (2) | (3) | (4) | (5) | (6) | (7) |
| --- | --- | --- | --- | --- | --- | --- | --- |
|  | **Internalizing disorder** | **Depression** | **Anxiety** | **Stress** | **Substance use disorder** | **Alcohol** | **Narcotics** |
| **Full sample** | | | | | | | |
| Earlier grades reform | 0.00244* | 0.0020** | 0.0015* | 0.0000 | 0.0008* | 0.0015*** | -0.0006** |
|  | (0.0013) | (0.0009) | (0.0009) | (0.0005) | (0.0005) | (0.0004) | (0.0003) |
| Observations: 524,093 |  |  |  |  |  |  |  |
| **Girls** | | | | | | | |
| Earlier grades reform | 0.0063*** | 0.0044*** | 0.0036** | 0.0001 | 0.0011 | 0.0017** | -0.0006 |
|  | (0.0021) | (0.0015) | (0.0015) | (0.0006) | (0.0009) | (0.0007) | (0.0005) |
| Observations: 254,901 |  |  |  |  |  |  |  |
| **Boys** | | | | | | | |
| Earlier grades reform | -0.0012 | -0.0003 | -0.0005 | -0.0002 | 0.0005 | 0.0013 | -0.0007 |
|  | (0.0014) | (0.0009) | (0.0010) | (0.0006) | (0.0010) | (0.0008) | (0.0005) |
| Observations: 269,192 |  |  |  |  |  |  |  |

Note: This table presents the estimated effects of earlier grading (in 6th versus 7th grade and 7th versus 8th grade) on the probability of receiving a mental disorder diagnosis in the year the student enters grade 9 of compulsory school. The results in each column and panel are from separate Difference-in-Discontinuities regressions, and the reported coefficients (ρ_2SLS_) represent the Local Average Treatment Effect of exposure to one year earlier grading. The sample includes all children born in Sweden within six months before and after January 1st in 1993, 1994, 1998, 1999, and 2000 (see Section 3 for detailed sample information). Robust standard errors clustered by birth month and year are reported in parentheses. ***p<0.01, **p<0.05, *p<0.1

**Table A5: Second-order polynomial specification – the effect of earlier grading on mental disorder diagnoses**

|  | (1) | (2) | (3) | (4) | (5) | (6) | (7) |
| --- | --- | --- | --- | --- | --- | --- | --- |
|  | **Internalizing disorder** | **Depression** | **Anxiety** | **Stress** | **Substance use disorder** | **Alcohol** | **Narcotics** |
| **Full sample** | | | | | | | |
| Earlier grades reform (incl 2^nd^) | 0.0035* | 0.0019 | 0.0028** | 0.0006 | 0.0030*** | 0.0035*** | -0.0004 |
|  | (0.0020) | (0.0016) | (0.0012) | (0.0008) | (0.0007) | (0.0007) | (0.0005) |
| Observations: 524,093 |  |  |  |  |  |  |  |
| **Girls** | | | | | | | |
| Earlier grades reform (incl 2^nd^) | 0.0054* | 0.0026 | 0.0044** | 0.0006 | 0.0045*** | 0.0049*** | -0.0001 |
|  | (0.0028) | (0.0024) | (0.0018) | (0.0011) | (0.0013) | (0.0010) | (0.0009) |
| Observations: 254,901 |  |  |  |  |  |  |  |
| **Boys** | | | | | | | |
| Earlier grades reform (incl 2^nd^) | 0.0018 | 0.0012 | 0.0013 | 0.0007 | 0.0014 | 0.0021 | -0.0007 |
| Observations: 269,192 | (0.0022) | (0.0014) | (0.0014) | (0.0009) | (0.0015) | (0.0014) | (0.0005) |

Note: This table presents the estimated effects of earlier grading (in 6th versus 7th grade and 7th versus 8th grade) on the probability of receiving a mental disorder diagnosis in the year the student enters grade 9 of compulsory school. The results in each column and panel are from separate Difference-in-Discontinuities regressions, and the reported coefficients (ρ_2SLS_) represent the Local Average Treatment Effect of exposure to one year earlier grading. The regressions include a second-order polynomial in the running variable (re-centered birth month). The sample consists of all children born in Sweden within six months before and after January 1st in 1993, 1994, 1998, 1999, and 2000 (see Section 3 for detailed sample information). Robust standard errors clustered by birth month and year are reported in parentheses. ***p<0.01, **p<0.05, *p<0.1

**Table A6: Reduced control sample (1998 cohort only) – the effect of earlier grading on mental disorder diagnoses**

|  | (1) | (2) | (3) | (4) | (5) | (6) | (7) |
| --- | --- | --- | --- | --- | --- | --- | --- |
|  | **Internalizing disorder** | **Depression** | **Anxiety** | **Stress** | **Substance use disorder** | **Alcohol** | **Narcotics** |
| **Full sample** | | | | | | | |
| Earlier grades reform (incl 2^nd^) | 0.00327 | 0.00215 | 0.00262 | 0.000527 | 0.00142* | 0.00182** | -0.000269 |
|  | (0.00231) | (0.00146) | (0.00192) | (0.000860) | (0.000775) | (0.000729) | (0.000599) |
| Observations: 524,093 |  |  |  |  |  |  |  |
| **Girls** | | | | | | | |
| Earlier grades reform (incl 2^nd^) | 0.00475 | 0.00379 | 0.00335 | 0.000652 | 0.00124 | 0.00179** | -0.000510 |
|  | (0.00386) | (0.00268) | (0.00291) | (0.00119) | (0.00110) | (0.000781) | (0.00101) |
| Observations: 254,901 |  |  |  |  |  |  |  |
| **Boys** | | | | | | | |
| Earlier grades reform (incl 2^nd^) | 0.00186 | 0.000495 | 0.00204 | 0.000385 | 0.00166 | 0.00194* | -1.99e-05 |
|  | (0.00253) | (0.00121) | (0.00202) | (0.000966) | (0.00113) | (0.00109) | (0.000813) |
| Observations: 269,192 |  |  |  |  |  |  |  |

Note: This table presents the estimated effects of earlier grading (in 6th versus 7th grade and 7th versus 8th grade) on the probability of receiving a mental disorder diagnosis in the year the student enters grade 9 of compulsory school. The results in each column and panel are from separate Difference-in-Discontinuities regressions, and the reported coefficients (ρ_2SLS_) represent the Local Average Treatment Effect of exposure to one year earlier grading. The sample consists of all children born in Sweden within six months before and after January 1st in 1998, 1999, and 2000 (see Section 3 for detailed sample information). Robust standard errors clustered by birth month and year are reported in parentheses. ***p<0.01, **p<0.05, *p<0.1

**Table A7: Alternative bandwidth – The effect of earlier grading on mental disorder diagnoses**

|  | (1) | (2) | (3) | (4) | (5) | (6) | (7) |
| --- | --- | --- | --- | --- | --- | --- | --- |
|  | **Internalizing disorder** | **Depression** | **Anxiety** | **Stress** | **Substance use disorder** | **Alcohol** | **Narcotics** |
| **Full sample** | | | | | | | |
| Earlier grades reform, August to May | 0.0029* | 0.0011 | 0.0021* | 0.0005 | 0.0018*** | 0.0023*** | -0.0004 |
|  | (0.0017) | (0.0010) | (0.0013) | (0.0006) | (0.0006) | (0.0006) | (0.0005) |
| Earlier grades reform, September to April | 0.0024 | 0.0017 | 0.0017 | 0.0010 | 0.0021*** | 0.0026*** | -0.0004 |
|  | (0.0019) | (0.0012) | (0.0014) | (0.0007) | (0.0005) | (0.0005) | (0.0005) |
| **Girls** | | | | | | | |
| Earlier grades reform, August to May | 0.0051* | 0.0023 | 0.0032 | 0.0006 | 0.0023** | 0.0029*** | -0.0002 |
|  | (0.0028) | (0.0018) | (0.0021) | (0.0009) | (0.0010) | (0.0007) | (0.0007) |
| Earlier grades reform, September to April | 0.0049 | 0.0027 | 0.0037* | 0.0017* | 0.0034*** | 0.0040*** | -0.0005 |
|  | (0.0030) | (0.0021) | (0.0022) | (0.0010) | (0.0010) | (0.0008) | (0.0008) |
| **Boys** | | | | | | | |
| Earlier grades reform, August to May | 0.0008 | -0.0001 | 0.0011 | 0.0004 | 0.0013 | 0.0018* | -0.0005 |
|  | (0.0020) | (0.0010) | (0.0013) | (0.0007) | (0.0010) | (0.0010) | (0.0006) |
| Earlier grades reform, September to April | 0.0001 | 0.0008 | -0.0001 | 0.0002 | 0.0010 | 0.0012 | -0.0002 |
|  | (0.0019) | (0.0012) | (0.0014) | (0.0008) | (0.0010) | (0.0010) | (0.0006) |

Note: This table presents the results for the impact of earlier grades (in 6th vs 7th and 7th vs 8th grade) on the probability of mental illness in the year the child enters 9th grade in compulsory school. The results in each column and panel are from separate Difference-in-Discontinuities regressions; the effect estimates (ρ_2SLS_) thus represent the Local Average Treatment Effect of exposure to one year earlier grading. The sample consists of all children born in Sweden four to five months before and after January 1st in 1993, 1994, 1998, 1999, and 2000 (see Data section for specific information about the sample). Clustered robust standard errors by birth month and year are reported in parentheses. ***p<0.01, **p<0.05, *p<0.1.

|  |
| --- |

**Figure A3: Frequency of births by month**

Note: This figure shows the mean number of births by month, from July through June, for the reform cohorts (children born six months before and after January 1st in 1999 and 2000) and the control cohorts (children born six months before and after January 1st in 1993, 1994, and 1998). For cohort definitions and sample details, see Section 3.

|  |  |
| --- | --- |
|  |  |
|  |  |

**Figure A4: Discontinuity graphs – covariate balance by month of birth**

Note: This figure shows standardized means and fitted linear predictions (including 95% confidence intervals clustered by birth month and year) for selected predetermined characteristics by month of birth, from July to June. Estimates are shown separately for the pooled control cohorts (children born six months before and after January 1st in 1993, 1994, and 1998) and the pooled reform cohorts (born around January 1st in 1999 and 2000). For sample details, see Section 3.

**Table A8: Covariate balance tests – the effect of earlier grading on predetermined characteristics**

|  | (1) | (2) | (3) | (4) | (5) | (6) |
| --- | --- | --- | --- | --- | --- | --- |
|  | **Foreign-born** | **Foreign-born parents** | **Low educated parents** | **Moderately educated parent** | **High educated parent** | **Parents’ income** |
| **Girls** | | | | | | |
| Earlier grades reform | 0.00908 | 0.0117* | 0.00329 | 0.0125 | -0.0158 | 4,357 |
|  | (0.00555) | (0.00610) | (0.00401) | (0.00980) | (0.00976) | (5,762) |
| Constant | 0.0960*** | 0.127*** | 0.0470*** | 0.507*** | 0.446*** | 255,603*** |
|  | (0.00248) | (0.00281) | (0.00185) | (0.00454) | (0.00450) | (1,944) |
| Observations | 254,901 | 254,901 | 254,901 | 254,901 | 254,901 | 241,393 |
| **Boys** | | | | | | |
| Earlier grades reform | 0.00126 | 0.00447 | 0.00518 | -0.00425 | -0.000929 | 5,452 |
|  | (0.00541) | (0.00598) | (0.00393) | (0.00966) | (0.00961) | (4,819) |
| Constant | 0.0887*** | 0.124*** | 0.0445*** | 0.515*** | 0.440*** | 253,822*** |
|  | (0.00240) | (0.00281) | (0.00185) | (0.00456) | (0.00452) | (1,914) |
| Observations | 269,192 | 269,192 | 269,192 | 269,192 | 269,192 | 255,008 |

Note: This table presents the results for the impact of earlier grades (in 6th vs 7th and 7th vs 8th grade) on several predetermined characteristics. Each column and panel report results from separate Difference-in-Discontinuities regressions; the estimated coefficients (ρ_2SLS_) represent the Local Average Treatment Effect exposure to one year earlier grading. The sample includes all children born in Sweden six months before and after January 1st in 1993, 1994, 1998, 1999, and 2000 (see Section 3 for detailed sample information).
Robust standard errors clustered by birth month and year are reported in parentheses. ***p<0.01, **p<0.05, *p<0.1.

|  |
| --- |

**Figure A5: Placebo reform exposure – effect on mental disorder diagnoses**

Note: This figure shows the estimated effect of placebo exposure to earlier grading on the probability of receiving a mental disorder diagnosis in the calendar year the student enters grade 9. Each marker represents the treatment effect estimate from separate difference-in-discontinuities regressions (ρ_2SLS_, as in Table 2). Spikes represent 95% confidence intervals clustered by birth month and year. The sample includes children born in Sweden six months before and after January 1st in 1993, 1994, and 1998 (see Section 3 for sample details).

**Table A9: Mechanisms – the effect of earlier grading on neurodevelopmental and behavioral diagnoses**

|  | (1) | (2) | (3) | (4) |
| --- | --- | --- | --- | --- |
|  | **Any**  **ICD-10 F90-98** | **ADD/ADHD** | **Conduct disorder** | **Autism** |
| **Full sample** | | | | |
| Earlier grades reform | -0.0015 | -0.0013 | 0.0000 | -0.0010 |
|  | (0.0013) | (0.0012) | (0.0005) | (0.0007) |
| Baseline | 0.0141 | 0.0114 | 0.0011 | 0.0041 |
| Observations: 524,093 |  |  |  |  |
| **Girls** | | | | |
| Earlier grades reform | -0.0003 | -0.0001 | -0.0008 | -0.0002 |
|  | (0.0019) | (0.0017) | (0.0006) | (0.0011) |
| Baseline | 0.0091 | 0.0066 | 0.0009 | 0.0028 |
| Observations: 254,901 |  |  |  |  |
| **Boys** | | | | |
| Earlier grades reform | -0.0025 | -0.0025 | 0.0006 | -0.0017 |
|  | (0.0022) | (0.0022) | (0.0007) | (0.0014) |
| Baseline | 0.0189 | 0.0159 | 0.0014 | 0.0054 |
| Observations: 269,192 |  |  |  |  |

Note: This table presents the results for the impact of earlier grades (in 6th vs 7th and 7th vs 8th grade) on the probability of receiving a mental disorder diagnosis in the calendar year the student enters grade 9 in compulsory school. Each column and panel report results from separate Difference-in-Discontinuities regressions; the estimated coefficients (ρ_2SLS_) represent the Local Average Treatment Effect of exposure to one-year earlier grading. The "Baseline" row shows the mean diagnosis rate in the control cohorts. The sample includes all children born in Sweden six months before and after January 1st in 1993, 1994, 1998, 1999, and 2000 (see Section 3 for detailed sample information). Robust standard errors clustered by birth month and year are reported in parentheses. ***p<0.01, **p<0.05, *p<0.1.
